# Deep mining of the protein energy landscape

**DOI:** 10.1063/4.0000180

**Published:** 2023-04-27

**Authors:** A. Joshua Wand

**Affiliations:** Departments of Biochemistry & Biophysics, Cell Biology & Genetics and Chemistry, Texas A&M University, College Station, Texas 77843, USA

## Abstract

For over half a century, it has been known that protein molecules naturally undergo extensive structural fluctuations, and that these internal motions are intimately related to their functional properties. The energy landscape view has provided a powerful framework for describing the various physical states that proteins visit during their lifetimes. This Perspective focuses on the commonly neglected and often disparaged axis of the protein energy landscape: entropy. Initially seen largely as a barrier to functionally relevant states of protein molecules, it has recently become clear that proteins retain considerable conformational entropy in the “native” state, and that this entropy can and often does contribute significantly to the free energy of fundamental protein properties, processes, and functions. NMR spectroscopy, molecular dynamics simulations, and emerging crystallographic views have matured in parallel to illuminate dynamic disorder of the “ground state” of proteins and their importance in not only transiting between biologically interesting structures but also greatly influencing their stability, cooperativity, and contribution to critical properties such as allostery.

## INTRODUCTION

The exquisite detail at the atomic scale of protein molecules derived from cryogenic x-ray crystallography^1^ has provided a powerful foundation for the development of structure–function relationships in proteins and has led to revolutionary advances in our understanding of how proteins actually “work.” Yet, it has been known, primarily from early spectroscopic, e.g.,^2^ and hydrogen exchange^3,4^ studies, at a time when the first of such structures were just being determined, that protein molecules are inherently dynamic. Indeed, the fundamental idea that proteins must “know” how to fold from the highly disordered unfolded state has driven many aspects of the field of protein biophysics.^5,6^ The protein folding problem leads naturally to the “funnel” in an almost tautological sense: if the unfolded state is highly disordered and the folded native state is not, then the configurational energy surface describing folding must be funnel-like. The key ingredient is what that surface looks like^7^ and how its shape and distinguishing features are created and ultimately influence the biological activity of protein molecules.^8,9^ The energy vs configurational (conformational) entropy diagram provides that framework, and the idea of “minimal frustration” leads to descriptive machinery that provides ways to incorporate a physics-based analysis.^10–12^ The concepts of the so-called wet and dry “molten globule” intermediates,^13,14^ contact order,^15^ defined (or not) folding pathways and the principles underlying them,^9,16,17^ the relative importance of the various forces governing protein stability and folding,^8^ and so on are all, in principle, unified by the energy landscape view.

Ironically perhaps, from the point of view of the residual entropy of protein molecules, it has turned out that the highly disordered unfolded state has arguably been more successfully characterized than the more structured states of proteins. Because of extensive averaging of various properties across the ensemble of structures comprising the unfolded state, both experiment and theory have led to rather satisfying descriptions.^18–20^ The idea of “roughness” of the energy landscape then leads conveniently to descriptions of nucleation and condensation of more definitive structure during folding, but even there, the multiplicity of “pathways” remains a somewhat contentious discussion (cf. Eaton and Wolynes^16^ and Englander and Mayne^21^) that can seemingly only be resolved by further experiment. Notwithstanding continuing disagreement about the extent of pathway multiplicity,^22^ the concept of sequential stabilization^23^ of small cooperative units of structure^4,9,24,25^ provides a simple and direct explanation of how proteins know to resolve the Levinthal paradox^6^ and fold to the ensemble of states that ultimately compose the functional properties of proteins.

The funnel of the energy landscape is often artistically rendered as having a sharp lowest energy state with accordingly little residual conformational entropy. This is often misread to mean something quite incorrect. The essential detail often overlooked by the casual reader is where kT crosses the funnel. The thermal energy is, of course, what ultimately defines to what degree various states of the protein will be occupied. Over the past decade or so, it has become apparent that kT is surprisingly high up the energy landscape, and that proteins retain a considerable amount of conformational entropy at physiological temperature. This conclusion comes largely from solution nuclear magnetic resonance (NMR), which is not only uniquely positioned to provide experimental evidence of internal motion but also of the attendant entropy that this motion represents.^26,27^ The predominant way to interpret NMR relaxation phenomena depending on fast motion of an NMR probe is through the so-called Lipari–Szabo squared generalized order parameter, which effectively quantifies the angular disorder of the NMR probe within the molecular frame of the protein.^28^ In a pioneering effort, Palmer and co-workers utilized a specific energy potential to make a connection between the dynamical behavior of protein backbone, as revealed by ^15^N relaxation, and the free energy of ligand binding.^29^ Subsequently, Yang and Kay^30^ and Wand and co-workers^31^ independently adopted this idea to make a connection of internal protein motion with conformational entropy. The idea is that motion between states can act as a proxy for the entropy representing the distribution across the interconverting states.^30,31^ In its original formulations,^30,31^ a specific isolated motional model (energy potential) was required, which is an obvious limitation. Though important insights were derived from motion of the protein backbone, e.g.,^32^ it subsequently became clear that most of the conformational entropy “action” resides in the motion of side chains.

For technical reasons, the NMR relaxation phenomena employed to measure motion require isolation of what will be a limited number of NMR probes within the protein.^26,27^ To overcome the incompleteness of the dynamical characterization and to avoid the obvious complications of specifying the energy potential governing the motion, an empirical approach was developed.^27,33^ It was noted that, though different energy potentials give different absolute entropies, changes in motion measured by NMR relaxation are relatively linearly related to underlying changes in conformational entropy.^31,34^ Furthermore, a simple formulation allowed the empirical calibration of a linear relationship between changes in fast side chain motion measured by NMR relaxation and changes in conformational entropy of the *entire* protein upon a change in functional state (e.g., the binding of a ligand).^27,33^ This is because the empirical calibration was constructed to also report on motion (entropy) of unmeasured sites in the tightly packed protein that are dynamically coupled to measured sites.^27,35^

**FIG. 1.**
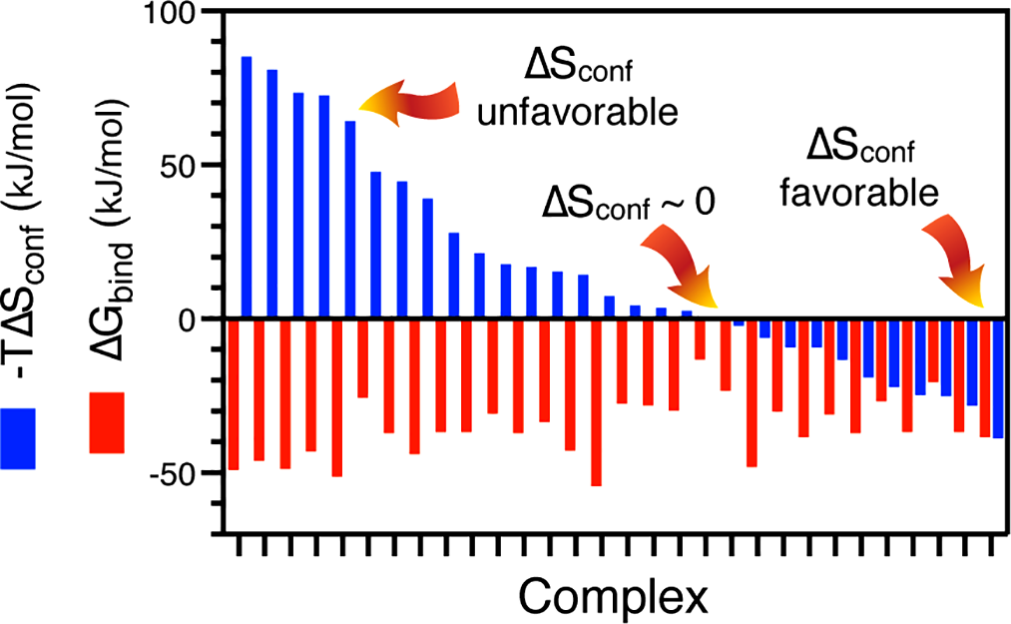
Contribution of protein conformational entropy to the free energy of ligand binding. The broad range of contributions available to proteins for high affinity binding of ligands is illustrated by the protein–ligand complexes used to calibrate the parameters of the entropy meter.^33^ Twenty-eight protein–ligand complexes and their isolated protein components were examined by NMR relaxation and methyl symmetry axis order parameters determined. These were used to calibrate the entropy meter. The resulting conformational entropies are arranged in descending order of the contribution of conformational entropy (blue bars) with the corresponding total free energy of binding measured by calorimetry (red bars). Conformational entropy contributed by the response of amino acid side chains to the binding of a ligand can vary from highly unfavorable to negligible to highly favorable. In some cases, conformational entropy is essential for high affinity binding (right side of distribution). The structural origins of the variable utilization of conformational entropy in molecular recognition are unknown. The extreme variability of the residual conformational entropy of stable proteins in their native state and its contribution to protein function was unanticipated. Redrawn from source data of Caro *et al.*^33^

Initial measurements with calcium-activated calmodulin revealed a remarkably variable dynamic response of methyl-bearing side chain motion upon high affinity binding of various peptides corresponding to minimal calmodulin-binding domains of regulated proteins.^36,37^ Various interpretations of this example suggested that conformational entropy can play a significant role in the thermodynamics of molecular recognition by proteins.^27,36–38^ This view was dramatically reinforced in the catabolite activating protein, where various point mutants illustrated the richness and sensitivity of the response of side chain dynamics, and the entropy it represents, to ligand binding.^39^ Subsequently, as the number of examples of protein–ligand interactions examined in this way grew to over two dozen high affinity protein–ligand complexes, the empirical “entropy meter” approach described above was developed.^33^ This construct showed that the response of proteins to ligand binding was quite variable with respect to conformational entropy.^33^ Indeed, perhaps counter-intuitively, the response of a protein upon binding a high affinity ligand can involve a large loss in conformational entropy that opposes binding, or result in a significant increase in conformational entropy to attain biologically meaningful affinity, or not contribute at all (Fig. 1).^33^ The “rules” governing this behavior are unknown though clues about its structural origins are now emerging.^40^ Furthermore, the variability of contributions of conformational entropy, which are largely invisible to classical structural methods (though see Fraser and co-workers^41–43^), unequivocally refutes the idea that one can assess the free energy of protein function simply by assessing the energy (often taken by pointing to features of a static structure) and ignoring the entropy as is generally the case in most analyses of structure–function relationships.

The interaction of protein molecules with those of solvent has been extensively pursued by experiment and theory. It now seems clear that the relatively weak water–protein interactions nominally associated with the “hydrophobic effect” do not influence significantly the conformational entropy of proteins at physiological temperatures.^44^ On the other hand, more long-lived specific interactions remain a challenge to characterize.^45^ Finally, it is interesting to note that recent studies indicate that integral membrane proteins have distinct side chain motion that is characterized by more extensive side chain rotameric averaging than their soluble protein colleagues.^46,47^ This helps explain why integral membrane proteins are stably folded in the membrane in the absence of the aforementioned hydrophobic effect—they have evolved to simply not give up as much conformational entropy upon folding as soluble proteins do. Nevertheless, it remains to be seen how the excess conformational entropy retained by membrane proteins influences other properties and functions such as ligand binding.

Proteins are exquisitely clever machines and do amazing things. Even “simple” functions such as ligand binding are difficult to rigorously describe.^48^ In addition to supporting chemical catalysis, proteins often display the cooperative coupling of binding of ligands.^49^ Termed allostery, this phenomenon is at the root of complex biochemistry, where molecular signals are integrated and transduced into biological action to remarkable effect. Allosteric regulation occurs in many contexts. Human adult hemoglobin (Hb A) is the classic example of the exquisite control of protein function through homo- and heterotropic allosteric regulation of ligand binding. Initially formulated as a strict two-state phenomenon where one state, termed the “relaxed” or “R” state, binds the molecular oxygen ligand with higher affinity than the other state termed the “tense” or “T.”^50^ However, the discovery of a second R-state^51^ being dynamically averaged with the original R-state in solution forever banished the idea of singular structures as being sufficient to describe allostery.^52^ Indeed, the modern treatment of allosteric regulation has become quite diverse and relies heavily on various aspects of the protein ensemble that are embodied in the energy landscape view.^53,54^ For example, the principle of frustration finds its expression in the mysterious agonist–antagonist switching behavior.^55^ A similarly rigorous statistical thermodynamic treatment established that an intrinsically disordered domain (IDD) in a protein could enhance its allosteric behavior through inter-domain coupling^56^ or the reciprocal effect, where a ligand to an IDD could either positively or negatively (depending on the sign of the coupling) tune the affinity of a coupled domain for another ligand.^57^ The full extent and variability of conformational entropy—represented by the breadth of the native state ensemble—is largely undocumented and ripe for mining. Indeed, the obvious connection of these types of phenomena to molecular evolution of proteins remains to be explored, especially in the context of life in extreme environments where temperature and pressure often exceed the parameters of current theoretical and experimental treatments.^58^

## Data Availability

Data sharing is not applicable to this article as no new data were created or analyzed in this study.
